# Assembly of *Olivibacter* sp. Strain UJ_SKK_5.1 Genome Sequence, Isolated from Metagenomes of *Macrotermes bellicosus* Guts Collected from Hot, Arid Nigeria

**DOI:** 10.1128/mra.00895-22

**Published:** 2022-10-06

**Authors:** Richard J. Kutshik, Bitrus Yakubu, Enoch B. Joel, Jessica L. Lenka, Aminu Tukur, Ishaya Y. Longdet

**Affiliations:** a Department of Biochemistry, University of Jos, Jos, Nigeria; b Biotechnology Centre, National Veterinary Research Institute, Vom, Nigeria; c Quality Control Dept, Nigeria National Petroleum Corporation, Port Harcourt Refinery, Alesa Eleme, Nigeria; University of Southern California

## Abstract

The metagenome-assembled genome sequence of *Olivibacter* sp. strain UJ_SKK_5.1 was generated from the metagenome of a Macrotermes bellicosus (termites) gut collected from Nigeria’s hot, arid environment. The assembled genome (6,135,249 bp) contains 432 contigs, with an *N*_50_ value of 22,779 bp, GC content of 41.1%, 5,043 protein-coding sequences, 5,034 proteins with functional assignments, and 9 pseudogenes and 48 RNA genes.

## ANNOUNCEMENT

The genus *Olivibacter*, belonging to the family *Sphingobacteriaceae*, was proposed less than 2 decades ago (1). *Olivibacter* species have been isolated from hydrocarbon-contaminated sites (2); dichlorodiphenyltrichloroethane (DDT)-contaminated soil (3); and clinical samples (4) displaying unique capacities, including ginsenoside conversion (5), diphenol degradation (6), hydrocarbon-degradation (2), and lignocellulose degradation (7). Here, we report the genome sequence of *Olivibacter* sp. strain UJ_SKK_5.1 derived from Macrotermes bellicosus guts.

*Macrotermes bellicosus,* collected from Illela, Sokoto State, Nigeria (latitude, 38.973166; longitude, 122.72809) in February 2021, was cleaned 3 times (dipped in 70% ethanol for 3 min and rinsed with sterile water). The extracted guts were crushed in phosphate-buffered saline (PBS). We were interested in organisms with a lignocellulose-degrading capacity. So, a portion of the crushed guts was cultured onto plated media prepared from kraft lignin, M9 salts, and agar in a ratio of 2:1:2 at 37°C for 72 h and subcultured in four successions on the same media. We thought colonies were pure isolates, but Gram staining revealed a mixture of organisms with varied morphological features (cocci, rods, pairs, chains, or single). To obtain cell pellets, the mixed organisms (from one colony) were grown overnight in nutrient broth at 37°C. Two sets of 2-mL portions (from the overnight broth) were centrifuged at 14,000 × *g* for 3 min. The combined cell pellets were washed (in 500 μL of phosphate-buffered saline and centrifuged at 14,000 × *g* for 3 min) twice.

Cell DNA was extracted using the ZymoBiomics DNA Miniprep kit according to the manufacturer’s instructions. DNA libraries were prepared using the Nextera XT DNA library preparation kit (Illumina) and the Nextera index kit (Illumina), and genomic DNA was fragmented using the Illumina Nextera XT fragmentation enzyme. Combinatory dual indexes were added to each sample followed by 12 cycles of PCR to construct libraries which were purified using AMpure magnetic beads, eluted in Qiagen EB buffer, quantified using a Qubit 4 fluorometer and Qubit double-stranded DNA (dsDNA) high-sensitivity (HS) assay kit, and sequenced on the Illumina HiSeqX platform with 2 × 150-bp read lengths producing 11.321 million raw reads. Raw metagenomic sequencing reads were trimmed and processed using Fastp v0.20.1 (8) with a cut mean quality of 15. The reads were assembled into contigs using MEGAHIT v1.0 (9) and then were binned using MetaBAT2 v2.15 (10) producing 5 hits, but only one metagenome-assembled genome (ABHB2) obtained had completeness (≥50%) and contamination (≤10%) (11). Here, default parameters were used for all software. Taxonomic classification, completeness, and contamination were assessed using QUAST v4.4 (12) and BUSCO v5 (13).

The ABHB2 genome has 432 contigs (mean, 14,201.96 bp), an assembled size of 6,135,249 bp, completeness of 98.40%, fragmentation of 0.08%, missing portion of 0.80%, an *N*_50_ value of 22,779 bp, GC content of 41.1%, and BUSCO % score of C:122 (S:122, D:0), F:1, M:1, n:124. The genome was annotated using PGAP v6.1 (14) identifying 5,043 protein-coding sequences, 5,034 proteins with functional assignments, 9 pseudogenes, and 48 RNA genes.

**FIG 1:**
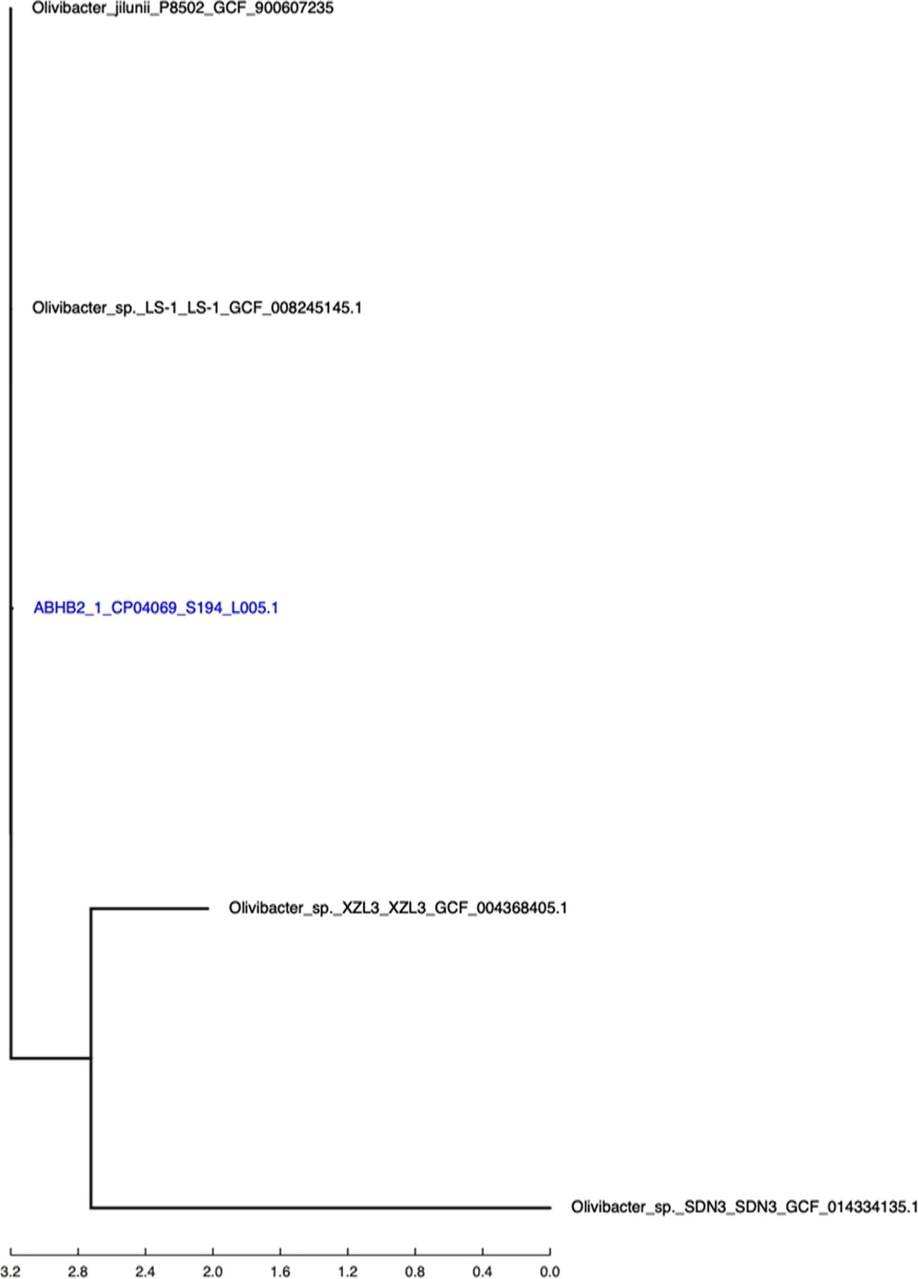
SNP tree based on core genome phylogeny of ABHB2.

The assembled contigs of ABHB2 genome were processed through CosmosID core genome single nucleotide polymorphism (SNP) typing pipeline to evaluate the phylogenetic placement and SNP differences using Parsnp (15) as the core genome aligner which reconstructed the phylogenomic relationship using FastTree2 (16). They generated a SNP tree (Fig. 1) revealing the closely related organisms as *Olivibacter_sp*._SDN3_SDN3 (GenBank accession number GCF_014334135.1), *Olivibacter_sp._*XZL3_XZL3 (GCF_004368405.1), *Olivibacter_sp*_LS-1_LS-1 (GCF_008245145.1), and *Olivibacter_jilunii*_P8502 (GCF_900607235.1). Therefore, we named the organism *Olivibacter* sp. strain UJ_SKK_5.1.

### Data availability.

The genome sequence for *Olivibacter* sp. strain UJ_SKK_5.1 has been deposited at DDBJ/EMBL/GenBank under accession number JAMDXX000000000. The version described in this paper is the first version, JAMDXX010000000. The raw reads were deposited in Sequence Read Archive (SRA) under accession number SRR19201141 and in BioSample under number SAMN28178924.
